# Wicking assisted condenser platform with patterned wettability for space application

**DOI:** 10.1038/s41598-023-45294-x

**Published:** 2023-10-23

**Authors:** Tibin M. Thomas, Pallab Sinha Mahapatra

**Affiliations:** Department of Mechanical Engineering, Indian Institute of Technology Madras, Chennai, 600036 India

**Keywords:** Mechanical engineering, Surface patterning, Surface chemistry

## Abstract

Vapor condensation is extensively used in applications that demand the exchange of a substantial amount of heat energy or the vapor-liquid phase conversion. In conventional condensers, the condensate removal from a subcooled surface is caused by gravity force. This restricts the use of such condensers in space applications or horizontal orientations. The current study demonstrates proof-of-concept of a novel plate-type condenser platform for passively removing condensate from a horizontally oriented surface to the surrounding wicking reservoir without gravity. The condensing surface is engineered with patterned wettabilities, which enables the continuous migration of condensate from the inner region of the condenser surface to the side edges via surface energy gradient. The surrounding wicking reservoir facilitates the continuous absorption of condensate from the side edges. The condensation dynamics on different substrates with patterned wettabilities are investigated, and their condensation heat transfer performance is compared. The continuous migration of condensate drops from a superhydrophobic to a superhydrophilic area can rejuvenate the nucleation sites in the superhydrophobic area, resulting in increased heat transport. The proposed condenser design with engineered wettability can be used for temperature and humidity management applications in space.

## Introduction

Condensation is an inevitable process in energy conversion^1^, environmental control^2^, water harvesting^3^, desalination^4^, and thermal management systems^5^. The overall efficiency of these systems has a greater dependence on the condensation process, and the performance of the condensation process is reliant on the mode of condensation, nucleation rate, and condensate removal rate^6–8^. These parameters are influenced by the surface wettability, which can be engineered by physical and chemical modifications^9–12^. The gravitational force plays a vital role in facilitating the removal of condensate from a surface, which is necessary for the continuous functioning of conventional condensers at optimum performance. The transport of condensate over the surface and the removal of condensate become challenging when a condenser surface is placed in a microgravity environment (space) or horizontal orientation^13^. In space applications, devices such as heat pipes^14,15^, porous media-based condensers^16^, and other complex condensing heat exchanger systems consisting of slurper bars and a rotary separator^17,18^ are used as a replacement for conventional condenser devices. Despite the fact that the wicking process ensures condensate transport via capillary forces, the fundamental disadvantage of these devices is their poor heat transfer performance due to film formation in the condensing area. Because the heat transfer performance of the filmwise condensation mode during pure vapor conditions is one order of magnitude lower than the dropwise mode^6^. Designing a plate-type condenser with superior performance that can be employed in space or horizontal orientation with either a dropwise mode or a mix of both filmwise and dropwise modes is challenging. The techniques used for self-transporting liquid on an open surface without gravity aid are important for developing condensers that can efficiently remove the condensate from the surface for space application.

Because of the exciting opportunities in micro-fluidics, lab-on-a-chip, condensation, and spray cooling applications, the self-transport of liquid on an open surface has attracted a lot of attention in the last decade^19–22^. Passive liquid transport on an open surface without gravity aid was accomplished through engineered surface modification techniques such as wettability gradient^23^, superhydrophilic wedge surrounded by a hydrophobic boundary^24^, topological liquid diode^25^, charge gradient^26^, and so on. On a wettability gradient surface, the difference in the Laplace pressure between two opposite ends drives the droplet from the high contact angle region to the low contact angle region^23^. Likewise, liquid transport occurs from the narrower end to the wider end of a superhydrophilic wedge track surrounded by superhydrophobic boundary due to the Laplace pressure difference between the front and back end^24,27^. The topological structure on the surface of a topological liquid diode breaks the contact line pinning of the drop at the advancing edge while simultaneously imparting a strong pinning force at the receding edge. This converts excess surface energy to kinetic energy, propelling the droplets from receding to advancing end^25^. On a charge gradient surface, droplets transport from a higher charge density region to a lower charge density region^26,28^.

In the presence of gravity, the condensate is removed from the vertically oriented surface when the size of the condensate reaches the capillary length scale, such that the gravity force of the condensate exceeds the surface tension forces^6^. Condensate droplets can also be removed at a size smaller than the capillary length scale via coalescence-induced droplet jumping from non-wettable surfaces^29,30^, periodic wicking structures from a hydrophilic surface^31,32^, and the application of an intermittent electrostatic potential to a hydrophilic surface^33^. In addition, various techniques of self-transport of liquid drops have been used in the condensation process to achieve directional transport of condensates or to improve overall condensation performance. On a surface with radially graded wettability, condensate was observed migrating from the central region of a substrate to the outer region^34,35^. Many researchers have also proposed bio-inspired structural graded structures that mimic spider silk, desert beetles, cactus spines, pitcher plants, and other natural materials for water harvesting from humid air via condensation and directional transport of condensate liquid^36–39^. When a superhydrophobic surface is infused with high viscous oil, condensate droplets can be seen levitating. The capillary force causes a meniscus to form over a condensate drop on an oil-infused surface. The stretching of the oil layer caused by droplet growth results in the droplets levitating^40^. Different types of surfaces with patterned wettability have also been proposed in the literature for directional condensate transport and to utilize the advantages of both hydrophilicity and hydrophobicity during condensation^41–45^.

**Figure 1:**
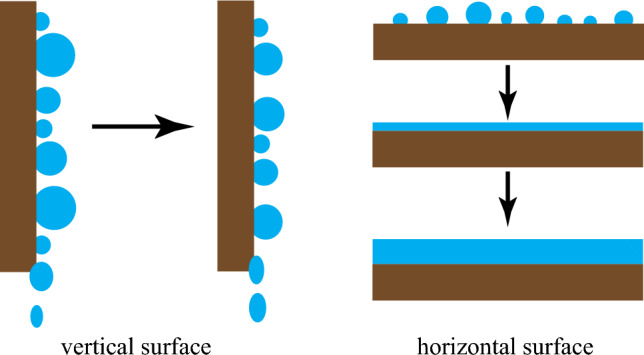
Schematic illustration of dropwise condensation on a vertical and horizontal oriented surface with time.

Previous research on condensation on a horizontally oriented substrate focused on either condensation mechanisms like nucleation, drop growth, and drop-coalescence, or passive droplet movement on the substrate with a radially smooth wettability gradient^34,35^ or a charge density gradient^28^. Nonetheless, it is yet unclear how to employ these substrates as condenser systems and how to continuously remove the migrating droplets from the substrate in a microgravity environment. Additionally, previous studies on the condensation process on a substrate with patterned wettability and the effectiveness of their condensation heat transfer primarily looked at vertically oriented or inclined substrates^41,42,46^. In such cases, the gravity force aids the removal of condensate from the substrate irrespective of the design of the various patterned wettabilities. On the other hand, when condensation occurs on a horizontal surface or a condenser surface kept in a microgravity environment, the condensate spreads and accumulates regardless of the substrate wettability. In such cases, removing condensate from the surface is challenging and condensate accumulates on the substrate with time, as shown in Fig. 1. To avoid liquid accumulation, condensate must be continuously removed from the surface using either passive or active techniques. Condensate can be removed in the microgravity environment by shearing airflow at the expense of energy^47,48^.

The current study proposes proof-of-concept for a plate-type condenser platform with patterned wettability as well as a novel passive method for the continuous removal of liquid formed by condensation on a horizontally oriented surface or the condenser surface in a microgravity environment via a wicking reservoir. A substrate with patterned wettability was designed to allow the passive transport of condensate droplets without external force or gravity. The gradient in surface energies propels the condensate formed on the substrate to the surface’s outer edges. The propelled condensate from the outer edges is absorbed by the wicking reservoir, which is surrounded by the substrate. Wherein, the wicking reservoir is not attached to the substrate, and a tiny gap is maintained between them to prevent heat conduction. The mechanism by which condensate is transported from the substrate to the wicking reservoir is explored. The condensation dynamics and condensation performance of four substrates with different patterned wettabilities at horizontal orientation are also investigated in this study. Additionally, differences in the droplet transport behaviors on the different patterned wettability surfaces have been investigated by steady-state numerical simulations and compared with experiments. The key findings of this study indicate that the early removal of condensate mass from a horizontal surface with optimal patterned wettability can maximize overall efficiency. Furthermore, a new approach to the experimental characterization of the condensation rate from a horizontally oriented substrate by a wicking reservoir was established in the present study.

## Results and Discussions

### Design of the patterned wettability and surface characterization

**Figure 2:**
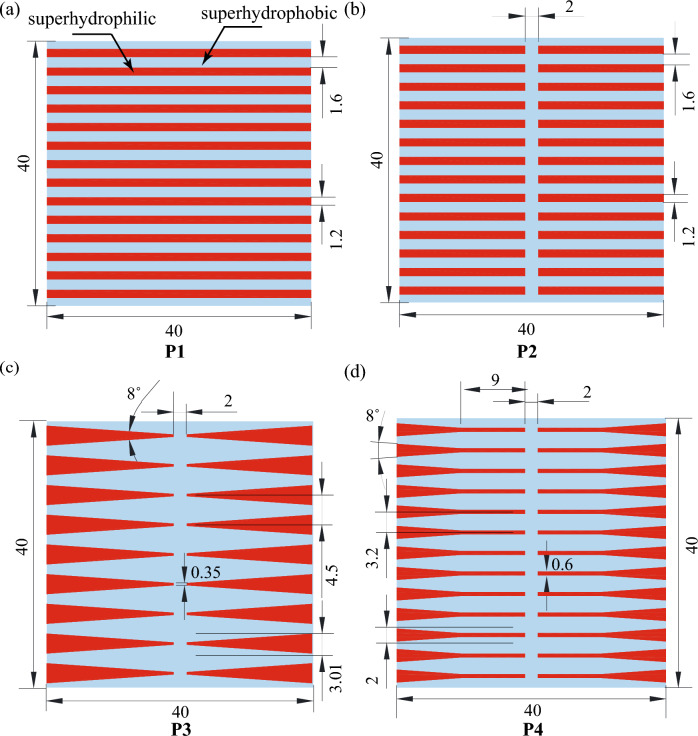
A plate type condenser substrate with patterned wettability comprising of SHL and SHB wettability regions (**a**) Pattern P1 - rectangular strip: full, (**b**) Pattern P2 - rectangular strip: half, (**c**) Pattern P3 - wedge, (**d**) Pattern P4 - hybrid (wedge + rectangular strip). The red shaded region represents the SHL area and the blue region represents the SHB area. Here, the SHB region promotes the dropwise mode of condensation and the SHL region promotes the filmwise mode of condensation. The SHL wedge tracks facilitate the transport of condensate from the inner region of the substrate to the side edge region. The units of all dimensions mentioned in this figure are in mm.

Figure 2 illustrates the design of the different substrates with patterned wettabilities used in the present study. The pattern P1 consists of fourteen parallel superhydrophilic (SHL) rectangular strips with a width of 1.2 mm and a length of 40 mm laid on a superhydrophobic (SHB) background (see Fig. 2a). Wherein, the spacing between the wettability transition lines of two consecutive SHL strips is set at 1.6 mm. For pattern P2, every SHL track in the pattern P1 is divided from the centre such that each of these tracks is converted to two rectangular tracks, as shown in Fig. 2b. Hence, the total number of SHL tracks became twenty-eight in the pattern P2 with a strip length of 19 mm. The central spacing between two in-line SHL strips is set at 2 mm to prevent the flooding in the centre of the substrate caused by the coalescence of condensate liquid from two opposed SHL wedges. In pattern P3, rectangular strips of pattern P2 were replaced with wedge-shaped tracks having a wedge angle of 8° (see Fig. 2c). The wedge angle of 8° is chosen in the design such that successful transport of a drop with a diameter greater than 1.8 mm (equal to the departure diameter for a hydrophobic surface in vertical orientation^49^) from the superhydrophobic area to the surrounding wicking reservoir is achievable once the drop has merged with the liquid at the narrower end of the wedge. The width of the wedge at its widest end was 3.01 mm, the narrower end was 0.35 mm and the pitch between two consecutive parallel wedges was 4.5 mm. In order to prevent droplet pinning at the narrower end, it is necessary to maintain a finite width at the narrower end. On pattern P3, the distance between the wettability transition lines of two consecutive SHL wedges ranges from 4.15 mm at the narrower end to 1.5 mm at the wider end. The minimum distance of 1.5 mm between the parallel wedges prevented liquid coalescence in the two subsequent SHL wedges. The total number of SHL tracks in the pattern P3 was reduced to eighteen compared to pattern P2 since the width of the tracks in the pattern P3 was increased to 3.01 mm compared to 1.2 mm. To increase the total number of SHL tracks, pattern P4 was designed by combining patterns P2 and P3. The various design parameters for pattern P4 were selected so that the total fractional area of the superhydrophilic track of the surface was kept in the range between 30–35%, like in pattern P3. Each SHL track in pattern P4 consists of a shorter wedge compared to the wedge in pattern P3, and the narrower end of the wedge was connected with a rectangular strip of width 0.6 mm and a length of 9 mm, as shown in Fig. 2d. The total number of SHL tracks on Pattern P3 increased to twenty-six. The width of the wider end of the wedge was 2 mm, the narrower end of the wedge was 0.6 mm, and the pitch between the two consecutive tracks was 3.2 mm. As a result, the distance between the wettability transition lines of two consecutive SHL tracks on pattern P4 was shortened compared to pattern P3, ranging from 2.6 mm at the narrower end to 1.2 mm at the wider end.

**Figure 3:**
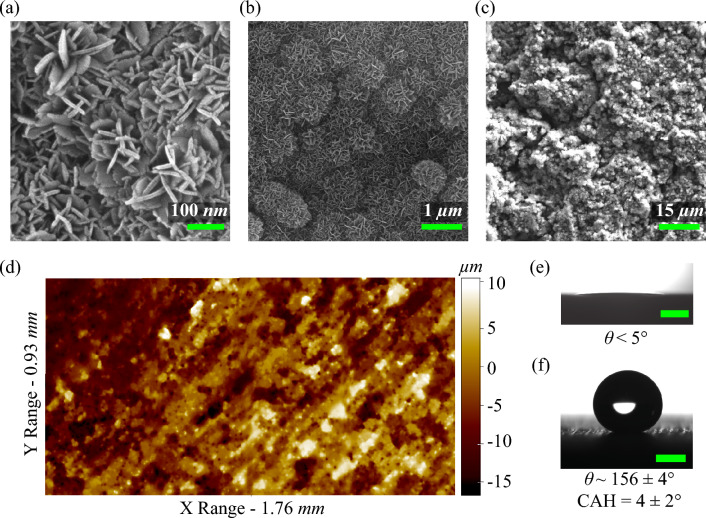
Surface morphology and wettability. (**a**–**c**) SEM, and (**d**) profilometer micrographs of the SHB surface. The first SEM image (**a**) shows blade-shaped nanostructures, whereas the second SEM micrograph (**b**) shows flower-shaped microstructures. Contact angle images of the water drop (**e**) before (SHL) and, (**f**) after hydrophobic functionalization (SHB) on textured aluminum substrate. The scale bars in the contact angle images are 1 mm.

Micro/nano textures on the aluminium surface were created by acid etching followed by hot water passivation^50^. The wettability of such surfaces becomes SHL, which can be further transformed to SHB by attaching hydrophobic molecules over the surface. Figure 3a,b,c shows the SEM images of the fabricated SHB surface at different resolutions. It is evident from the SEM micrographs that the modified SHB surface consists of $Al_2O_3$ blade-shaped nanostructures (see Fig. 3a) and flower-shaped microstructures (see Fig. 3b). The length and width of the blade-shaped nanostructures were in the range of 100–300 nm and 15-30 nm, respectively. The size of the flower-shaped microstructures was in the range of 0.5–1 μm. The physical structure of the SHB surface is identical to the surface morphology of the SHL surface before the silane functionalization since the self-assembled monolayer could only make a layer of negligible thickness of 30 Å^51^. The average macroscopic roughness of the textured surface was ~4.2 μm. The textures on the modified rough surface were random, and the peak height and valley depth were in the order of ~10 μm and ~15 μm respectively (see Fig. 3d). The contact angle of the sessile water droplet before the hydrophobic coating was nearly zero. The hydrophobic coating changed the surface wettability of the textured surface to superhydrophobicity, as shown in Fig. 3e,f. The magnitudes of the advancing ($\theta _A$) and receding ($\theta _R$) contact angles on the SHB surface were $159\pm 2^\circ$ and $155\pm 2^\circ$ respectively. The contact angle hysteresis (CAH) is defined as the difference between advancing and receding contact angles, and the magnitude of the CAH for the SHB surface was 4±2°. The SHL tracks on the substrate were fabricated by selective etching of the hydrophobic coating using a CO_2_ laser. As a result, the laser-exposed area on the substrate transitioned to superhydrophilic with a contact angle of below 5°.

### Wicking reservoir

**Figure 4:**
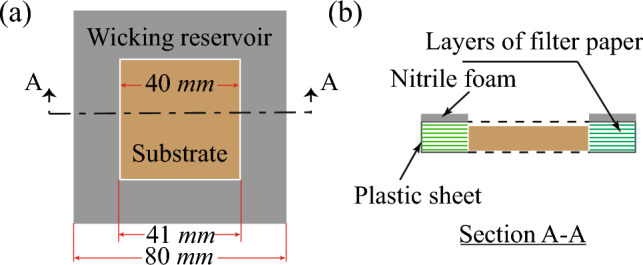
Design of the wicking reservoir (**a**) Illustration of wicking reservoir (gray colour) placed surrounded to the condensing substrate. To prevent heat transfer losses due to conduction between the wicking reservoir and the substrate, a gap of 0.5 mm was maintained. (**b**) Sectional view of wicking reservoir, comprising of layers of filter papers, plastic layer covering and nitrile foam insulation.

A wicking reservoir was used to passively remove the condensate from a horizontal substrate. The wicking reservoir is made up of multiple layers of Whatman filter papers of Grade 1 as interior layers and a thin plastic sheet as top and bottom layers, as shown in Fig. 4. Each layer of filter paper was cut according to the dimensions mentioned in Fig. 4a using a CO_2_ laser at a power setting of 5% of total power and 3 % of total speed. The laser-cut papers were then stacked, and the top and bottom sides of the stack were covered with a laser-cut plastic sheet of the same dimension using double-sided adhesive tape. To prevent mass loss of condensates collected from the substrate, the lateral sides of the wicking reservoir were covered with plastic tape. The top surface of the wicking reservoir was insulated with 3 mm of thick nitrile foam tape to prevent water vapour condensation on the wicking reservoir (see Fig. 4b).

### Condensation observation

**Figure 5:**
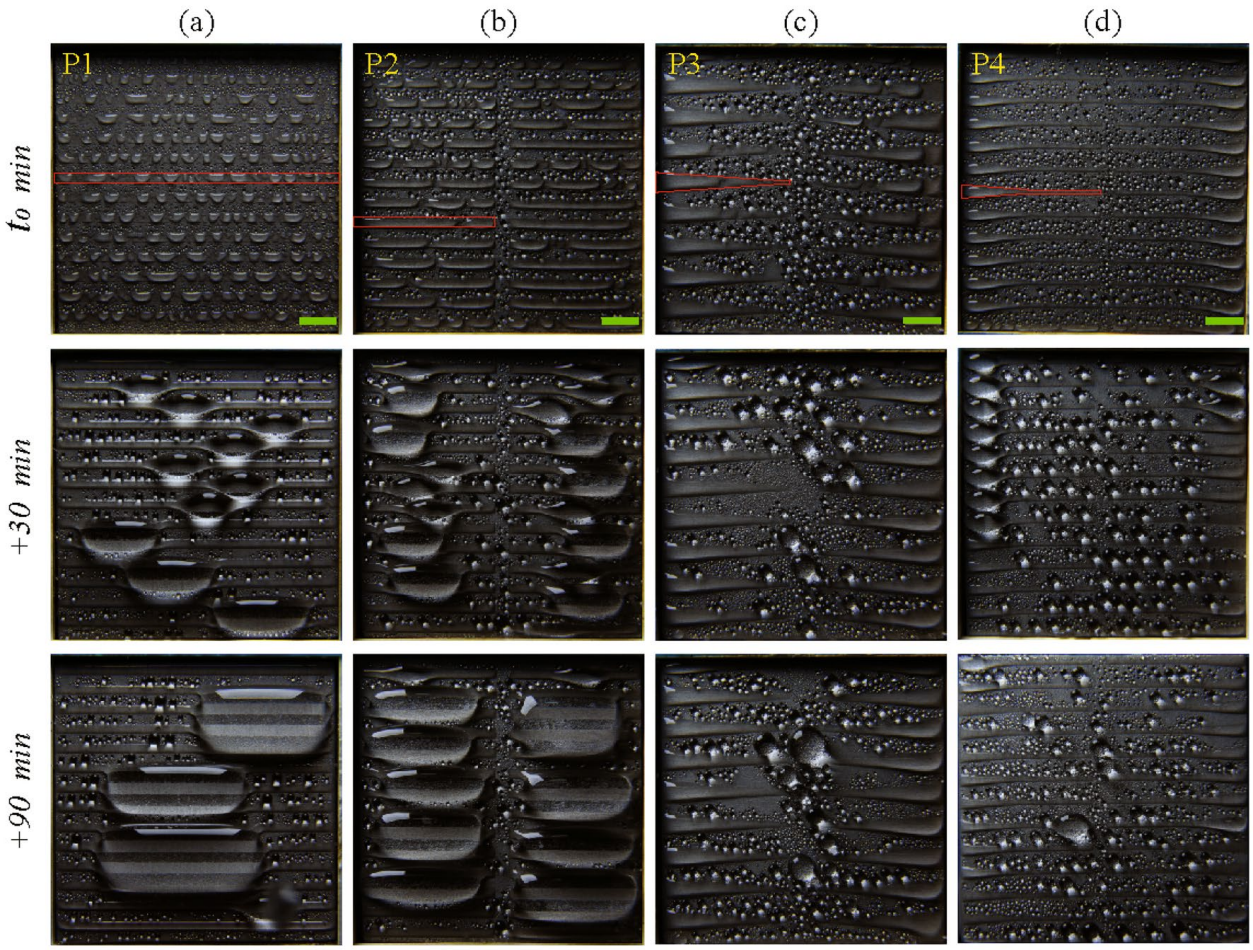
Macroscale condensation snapshots of different substrates with patterned wettability at different times. The red-coloured contour represents the shape of the SHL track in each patterned substrate. $t_0$ denotes the time when the temperature of the substrate reaches $T_s$. The scale bar is 5 mm.

Condensation is a complex phenomenon that consists of various processes such as drop nucleation, droplet growth, and coalescence^7^. Typically, condensate that forms on a surface drains away when the gravity force of the condensate becomes higher than the force of surface adhesion. However, in a microgravity environment, the generated condensate on a homogeneous surface coalesces and accumulates over the surface as multiple bulges, further transitioning to a thick film. The removal of condensate from a surface before the formation of a larger-sized liquid bulge is necessary for the continuous operation of a condenser at a desired heat transfer rate. The wicking reservoir of the proposed platform helps to remove the condensate from the substrate. The liquid-vapor interface of the condensate must overcome the gap between the substrate side edges and the wicking reservoir in order to start wicking. The condensate transport mechanism and its performance on four different substrates with patterned wettability in a controlled humid air environment are compared in this study. Figure 5 depicts the snap-shots of the condensation phenomena on the horizontally oriented substrates having different patterned wettabilities at an interval of 30 min. The experiments were carried out in an environmental chamber with a temperature ($T_{env}$) of 40 °C, a relative humidity (RH) of 80% and a substrate temperature ($T_s$) of 4±1 °C. At the initial condensation stage, the substrates showed a dropwise mode of condensation on the SHB area and a filmwise condensation mode on the SHL area. On pattern P1, the amount of condensate liquid increased in the rectangular SHL tracks and formed a liquid bulge with time since the condensate was not removed from the substrate. This liquid bulge grew with time, and the liquid interface of the bulge coalesced with either the neighbouring bulge or the neighbouring SHL rectangular track. After a few more coalescences, the liquid bulges over the centre region of the substrate and floods (see Fig. 5a). These liquid bulges prevent the effective spread of condensate through the SHL tracks and resist the continuous removal of condensate through the substrate side edges by wicking. The formation of larger sized liquid bulges was also observed on the substrate with pattern P2. The total volume and the total number of bulges were found to be higher on pattern P2 than on pattern P1. The central SHB area on pattern P2 avoids the merging of liquid bulges formed on the SHL tracks found on the opposite sides, as shown in Fig. 5b. Interestingly, flooding of the condensate was not observed on patterns P3 and P4 (see Fig. 5c,d). This indicates that the nucleated condensate was continuously removed from these substrates with patterned wettability P3 and P4.

**Figure 6:**
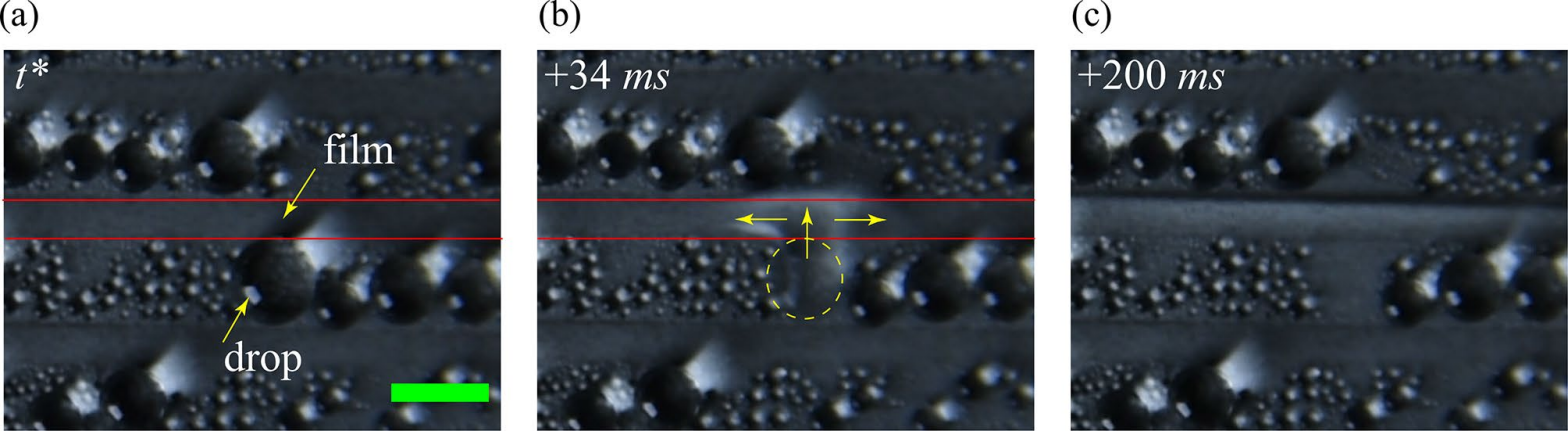
The coalescence between drop and film causes the capillary-driven pumping of condensate from the SHB region to the SHL region on pattern P1 and P2. The red solid line represents the transition line between the SHB and SHL areas. The yellow arrow line represents the liquid transport from drop to film. The scale bar is 3 mm.

Condensate appeared as distinct drops with different sizes in the SHB area. Whereas condensate was observed as a liquid film on the SHL tracks. The condensate drops from the SHB region were passively transported to the SHL tracks by coalescence-driven capillary pumping^42,46^. The nucleated drop in the SHB region grew with time, and it coalesced with the nearest condensate film in the SHL region once the liquid interface of the drop was in contact with the wettability transition line, as shown in Fig. 6. This liquid transport occurs due to the difference in the Laplace pressure acting at the liquid-vapor interface between the drop and the film. The higher interfacial Laplace pressure of the drop compared to the liquid film having an infinite radius of curvature causes liquid transport from the drop to the film in the SHL track. After the coalescence, the liquid-vapor interface of the liquid film in the SHL region crosses the wettability transition line (see Fig. 6c) due to inertia, and finally the liquid volume from the drop is spread out over the SHL track.

**Figure 7:**
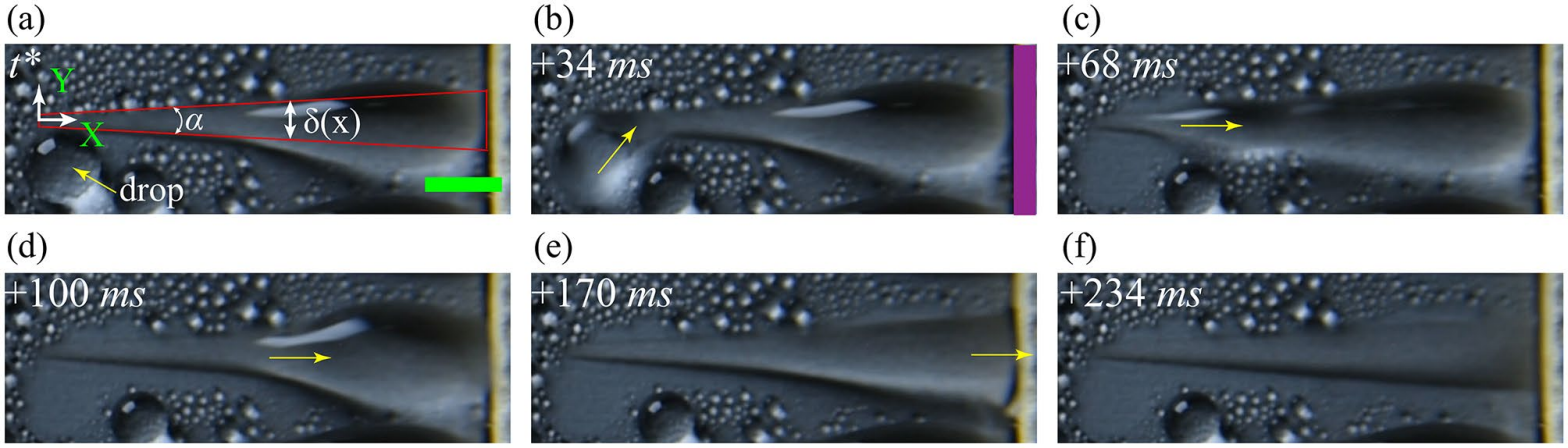
An illustration of the passive transport of condensate through an SHL wedge and the transport of condensate from substrate to wicking reservoir in the pattern P3. The red solid line represents the transition line between the SHB and SHL areas. The yellow arrow line represents the direction of liquid transport. The violet-coloured shaded region represents the side end of the wicking reservoir. The scale bar is 3 mm.

Figure 7 demonstrates the transport of a liquid drop from the SHB region to the wicking reservoir via the SHL wedge on pattern P3 during condensation. Wherein, the SHL track is completely filled with the condensate before the drop-film coalescence event. The drop from the SHB region is transported to the SHL region once the liquid interface of the drop is in contact with the liquid film present in the SHL track due to capillary pumping^42,46^. Thereafter, the liquid is transported inside the wedge track from the narrower end to the wider end by a Laplace pressure gradient arising due to the curvature difference between the front and back end^23,24^. The magnitude of the pressure gradient of the liquid bulge in the SHL track is calculated based on scaling arguments^24^,

1 $$\begin{aligned} \frac{dP}{dx}\approx 2\sigma _{lv}sin\theta _{avg}\frac{\alpha }{2\delta (x)^2} \end{aligned}$$

where $\sigma _{lv}$ is the interfacial surface tension, $\theta _{avg}$ is the average of the front and back end apparent contact angles of the liquid bulge, $\alpha$ is the included angle of the wedge track, and $\delta (x)$ is the track width of the wedge at a local position of *x* from the narrower end in the direction of liquid transport (see Fig. 7a). Wherein, the SHL track is not completely filled with the condensate prior to drop-film coalescence. The transported liquid accumulated in the wider region of the wedge due to the substrate edge resistance. The size of the accumulated liquid bulge inside the SHL track increases with time, and the liquid interface of the bulge merges with the surrounded wicking reservoir. Thereafter, the wicking reservoir absorbs condensate from the substrate by wicking.

**Figure 8:**
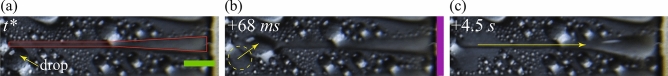
Illustration of liquid transport from the thin SHL rectangular track to the wider end of the wedge in the pattern P4. The red solid line represents the transition line between the SHB and SHL areas. The yellow arrow line represents the direction of liquid transport. The violet-coloured shaded region represents the side end of the wicking reservoir. The scale bar is 3 mm.

In pattern P4, the hybrid SHL track was designed by superimposing the wedge pattern on a rectangular striped pattern. The condensate transported to the rectangular stripe region from the SHB area spreads into the rectangular shaped region of the hybrid SHL track with time, as shown in Fig. 8. The transport of condensate is resisted at the merging region between the rectangular and wedge SHL tracks. The maximum accumulation in the rectangular region drives the transport of condensate from the rectangular region to the narrower region of the wedge. Further, the liquid from the narrower end is transported to the wider end by a difference in Laplace pressure, and the accumulated condensate in the wider region of the wedge is absorbed by the surrounded wicking reservoir, similar to pattern P3. The total volume of the liquid that can be accommodated within a single SHL track on pattern P3 is higher as compared to pattern P4.

The superhydrophilic tracks in the patterned substrate act as an intermediate reservoir before the condensate is transported to the wicking reservoir placed outside of the condenser plate. The drops from the superhydrophobic region can coalesce with the liquid film at different locations on the substrate. After the coalescence, the area occupied by the coalesced drop in the superhydrophobic region will become dry, and further drop nucleation will occur. The time scale of the liquid transport within the superhydrophilic track is depend on the volume of the condensate occupied in the track and the location of the drop when it connected with the wettability transition line. Condensate wicking from the substrate can occur only when the superhydrophilic track is completely filled with condensate and the liquid interface of the bulge bridges the gap between the substrate and wicking reservoir. The time scale for the transport process of the nucleated drops from the superhydrophobic region to the superhydrophilic region is influenced by the pitch between the superhydrophilic track and the total length of the wettability transition line. The total length of the wettability transition line was ~0.7 m, and ~1 m for the patterned surfaces P3 and P4, respectively. Hence, the probability for the occurrence of drop to film coalescence process is higher for pattern P4 than pattern P3 due to 42% increase in the total length of wettability transition line for the pattern P4.

### Droplet transport on wettability engineered superhydrophilic tracks

**Figure 9:**
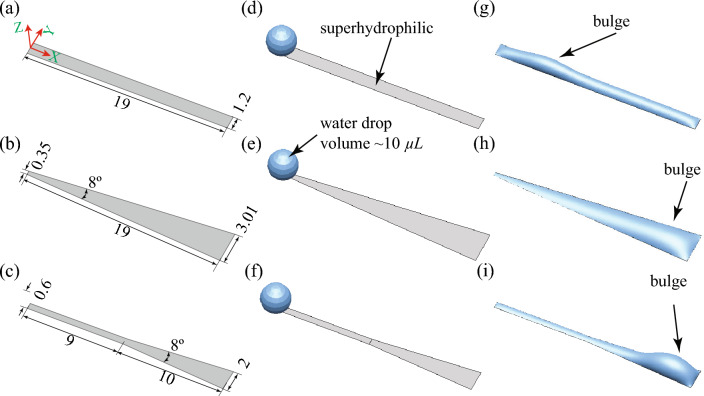
Steady state droplet shape on different patterned superhydrophilic tracks. (**a**–**c**) The shape and size of the various superhydrophilic track patterns (Patterns P2, P3, and P4, respectively). The wettability outside of the track is superhydrophobic. All dimensions are in mm. (**d**–**f**) The droplet’s initial condition on a patterned superhydrophilic rectangular track (pattern P2), wedge-shaped track (pattern P3), or combined rectangular and wedge track (pattern P4). (**g**) The final steady-state shape of the droplet on a rectangular track. The bulge is formed near the initial droplet location. (**h**) The droplet was transported to the end of the wedge track and the bulge formed near the wider end of the track. (**i**) The droplet was transported efficiently and a larger-sized bulge formed at the end of the track. The units of all dimensions mentioned in this figure are in mm.

To identify the differences in the droplet transport behaviors on the different patterned wettability surfaces, steady-state simulations are performed using the HyDro droplet simulation solver^52^. Matsui et al.^52^ developed HyDro using hybrid energy minimization techniques to identify the equilibrium shape of a droplet placed on a surface. In the simulations, only the transport of a single droplet on smooth patterned wettable surfaces is shown. The actual condensing scenarios are much more complex, with multiple droplet coalescence, droplet-to-film coalescence, and hierarchical surface roughness. To mimic the bulge formation on the superhydrophilic tracks observed during condensation, we have placed a droplet of volume 10 μL on different patterned wettable surfaces, as shown in Fig. 9. Here the superhydrophilic tracks are laid on a superhydrophobic background. Therefore, when a droplet is placed at the start of the track, the droplet spreads till the end of the track. However, the mechanism of droplet transport in the different tracks or patterns is different. The droplet spreads on the rectangular track (pattern P2) due to the surface’s high energy. After the liquid had spread to the end of the track, a bulge formed near the initial droplet location (see Fig. 9g). On the wedge track (pattern P3), the liquid spreads due to the net capillary force caused by the difference in curvature between the droplet’s front and back^53^. As the liquid is pumped due to the capillary force, the bulge formation occurs near the wider end of the track (see Fig. 9h). However, the bulge is spread over a longer distance. On the combined rectangular and wedge track (pattern P4), the droplet transport takes place initially on the rectangular track and then reaches the wedge region through the capillary transport on the wedge track. This transport is very efficient, as the liquid can move forward easily through the capillary transport but cannot go back to the rectangular track due to the requirement of extra pressure (working like a natural valve). Hence, the liquid bulge forms near the end of the wedge track (see Fig. 9i). The formation of liquid bulges on the various superhydrophilic tracks observed during experiments is identical to the results obtained from steady-state simulations (see Figs. 5, 7d, and 8c).

**Figure 10:**
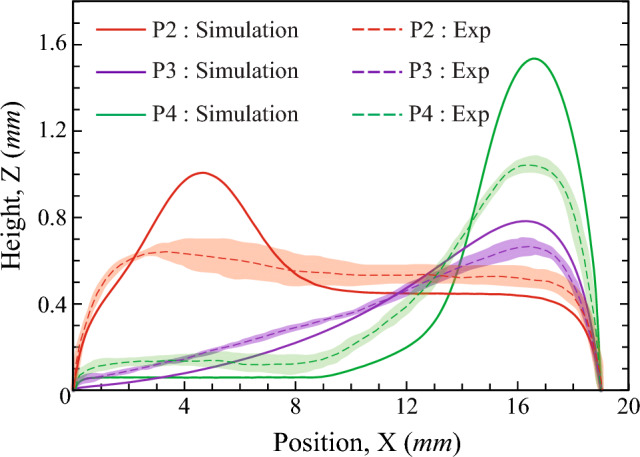
Numerical and experimental comparison of the variation in steady-state liquid height at the central plane ($Y=0$) of the different shaped patterns along the track length of the superhydrophilic track. $X=0$ mm represents the initial position of the drop and $X=19$ mm represents the side edge of the substrate. The shaded region accounts for the uncertainties in the measurements.

Figure 10 depicts the height of the liquid on the central plane ($Y = 0$) of the superhydrophilic track from the start ($X = 0$ mm) to the end ($X = 19$ mm) position obtained from simulation and experiments. The numerical model used in this study does not account for various parameters, like hierarchical roughness of the solid surface, heterogeneity of surface textures, roughness of the SHB-SHL boundary line, and so on. This led to hemi-wicking of the liquid placed on the SHL track as well as increased flow resistance due to heterogeneous rough textures. As a result, the numerical predictions differ from the experimental observations. The predicted maximum liquid bulge height from the simulation is higher than the experimental results. In contrast, the characteristic behaviour of numerical and experimental results is similar. In the simulation, for the case of pattern P2, the liquid height increases from the initial position and reaches a maximum value at $X=4.75$ mm, and further, the height reduces till $X=8.5$ mm. Thereafter, the liquid height is maintained constant, and a sharp reduction occurs at the end of the track. For pattern P3, a smooth increase in the liquid height was observed till $X=16.5$ mm, and it further reduced at the track end. The liquid height in the wedge region of the pattern P4 first increases up to $X=16.5$ mm and then reduces till the end of the track. Whereas, a thin liquid film forms on the rectangular region of the track. Due to the larger bulge at the end of the track, the liquid in the superhydrophilic track of pattern P4 can easily come into contact with the wicking material placed on the sides of the condenser plate.

### Condensation performance

**Figure 11:**
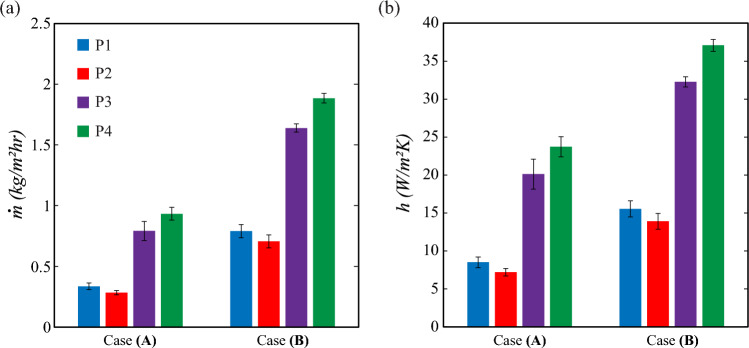
Comparison of condensation performance on different substrates with patterned wettability. (**a**) The average rate of condensate collection by the wicking reservoir on different substrates. (**b**) Average heat transfer coefficients of various substrates.

The condensation experiments on the horizontally oriented substrates were performed under two different conditions: Case (A) $T_{env}=30$ °C, RH= 80 %, and $T_s$= 1.5±0.5 °C, and Case (B) $T_{env}=40$ °C, RH= 80 %, and $T_s$= 4±1 °C. The dew point temperature of Case (A) is $T_{dew}=26.1$ °C and Case (B) is $T_{dew}=35.8$ °C. The difference in the mass of the wicking reservoir before and after the condensation experiments was calculated to compare the condensation performance of different substrates with patterned wettability. The pattern P2 showed the lowest, and pattern P4 showed the highest condensation removal rate in both environmental conditions, as shown in Fig. 11a. The formation of larger-sized liquid bulges observed on both sides of the pattern P2 substrate caused the delay in the condensate transport to the wicking reservoir. However, the wicking occurs when the liquid interface of the liquid bulge crosses the side edge of the substrate. Pattern P4 with hybrid SHL tracks has shown a better condensation removal rate than pattern P3 with wedge-shaped SHL tracks.

The effective condensation heat transfer coefficient (CHTC) of the substrate is defined as^42^,

2 $$\begin{aligned} h=\frac{{\dot{m}} h_{fg}}{T_{dew}-T_s} \end{aligned}$$

where ${\dot{m}}$ is the total condensate mass collected by the wicking reservoir, $h_{fg}$ is the latent heat absorbed by the substrate during condensation, $T_{dew}$ is the dew point temperature, and $T_s$ is the substrate temperature. The accurate collection of condensate without any mass losses is important for predicting the CHTC accurately. As the mass collection system is kept in a humid environment, evaporation of the collected condensate occurs and can lead to the underprediction of the CHTC. In the present study, condensate collection is achieved with a wicking reservoir, as shown in Fig. 4. Here, the wicking reservoir is covered with a plastic sheet on all the faces except the interior faces parallel to the substrate edges, through which the condensate is absorbed. Hence, evaporation occurs only through the faces that are parallel to the substrate edges. The temperature of the humid air near the interior faces of the wicking reservoir is much lower than the $T_{env}$ due to sensible cooling. Hence, the evaporation loss during condensate collection is negligible and the corresponding contribution to the heat transfer coefficient is not accounted for in Eq. (2).

The formation of a greater number of liquid bulges reduces the CHTC on pattern P2 by ~15% and ~10% compared to pattern P1 at the experimental conditions A and B, respectively (see Fig. 11b). The flooding of condensate was not observed in patterns P3 and P4. The passive transport of condensate through SHL wedge tracks on the patterns P3 and P4 prevented condensate flooding on the substrate. Hence, the condensation performance on substrates having patterns P3 and P4 was found to be better as compared to the substrates with patterns P1 and P2. In experimental condition A, the CHTC of pattern P3 and P4 substrates was enhanced by ~136% and ~178% compared to pattern P1. For the experimental condition B, the enhancement was ~107% and ~138%, respectively, for the substrates with pattern P3 and P4. Interestingly, the CHTC of pattern P4 was enhanced by ~18% and ~15% compared to pattern P3 at environmental conditions A and B, respectively. The main reasons for this enhancement were the increase in the number of SHL tracks and the decrease in the area occupied by the single SHL track on pattern P4 compared to pattern P3.

### Design limitations and scalability of the condenser platform

This study explored the feasibility of a plate-type condenser that can operate continuously in a humid air environment in microgravity. The current work attempted to address challenges related to removing condensate from the condenser substrate in a microgravity condition. The condensate is transported from the interior area of the horizontally oriented substrate to the side edges by passive transport in the wedge shaped patterned wettability, and its removal is aided by continuous wicking from the side edges by a wicking reservoir. However, the proposed condenser platform may fail once the wicking reservoir is saturated with the condensate collected from the substrate, which could further result in surface flooding. When the condenser is run continuously for a longer duration or under high condensation flux conditions, the wicking reservoir can saturate with the condensate liquid. Hence, the surrounded wicking reservoir can only act as an intermediate reservoir, and condensate must be continuously extracted from the wicking reservoir by an external system during long-term operation. This can be accomplished by making micrometer-sized holes in the wicking reservoir, with one end closed and the other end attached to a micrometer-sized capillary tube. When the wicking reservoir is filled with the condensate absorbed from the substrate, the holes in the reservoir begin to fill. Thereafter, the condensate will be carried by capillary action through the connected capillary tube. This capillary tube may continuously suck the collected condensate from the wicking reservoir. Furthermore, the condensate from this tube can be pumped to a storage tank using either a suction pump^16^ or a capillary ejection mechanism^54,55^ in microgravity.

**Figure 12:**
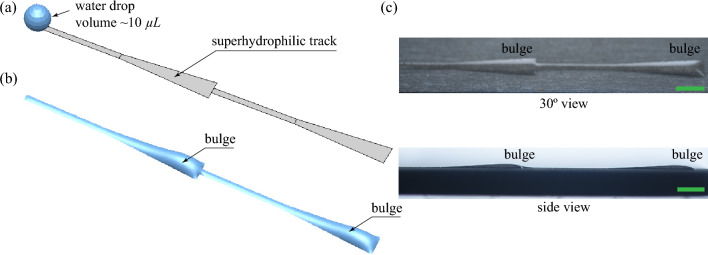
Steady-state simulation of the passive droplet transport in the two superhydrophilic tracks of pattern P4 that have been sandwiched in series. (**a**) Initial, and (**b**) final state from the simulation. (**c**) Experimental evidence of a droplet self-transporting from one superhydrophilic track to the sandwiched track on a horizontally oriented surface. During simulation and experimental testing, the volume of the water droplet was 10 μL. The scale bar is 3 mm.

The proposed condenser platform had an active condenser area of 40 × 40 mm^2^. During the scaling of the condenser area, the length of the superhydrophilic tracks should also be increased. However, the passive transport of the condensate may fail when the length of the wedge is higher, which can cause surface flooding. Therefore, the proposed patterned design must be modified for a larger substrate to avoid the flooding issue. In such cases, multiple superhydrophilic tracks in the pattern P4 can be sandwiched in series to increase the overall length of the superhydrophilic track, as shown in Fig. 12a. The steady-state simulation elucidated that the passive transport of the droplet is possible even with two superhydrophilic tracks that are connected in series (see Fig. 12b). In addition, experimental testing verifies the simulation-predicted behaviour of the droplet transport, as shown in Fig. 12c. For testing, a droplet of volume 10 μL was placed at the side edges of the rectangular-shaped superhydrophilic track using micro-pipette. The droplet initially spreads laterally across the rectangular track and reaches up to the narrower end of the first wedge. Due to the pressure gradient between the narrower and wider ends of the wedge, self-transport of liquid subsequently occurs. This pressure gradient can further push the liquid out of the wedge-shaped pattern P4 and enter the sandwiched rectangular-shaped track. Again, the liquid reaches the narrower portion of the following wedge track and is transported to the wider end of the wedge track. A liquid bulge was formed in the wider end of both wedges. The size of the liquid bulges will increase when the droplets from the superhydrophobic region are transported to the superhydrophilic track by drop-film coalescence. Further, the wicking at the side edges will occur when the interface of the liquid bulge close to the side edges exceeds the gap between the substrate and wicking reservoir.

A sufficient wettability contrast between the hydrophilic and hydrophobic regions is a primary requirement for the efficient transport of condensate through the superhydrophilic wedge track and the transport of drop from the hydrophobic region to the hydrophilic track^53,56^. The absorption of volatile organic compounds (VOCs) present in the atmosphere might raise the contact angle of the superhydrophilic region in ambient conditions^57^. However, such possibilities are rare on the space station because atmospheric air conditions are artificially created in the space station using purified gases carried from Earth. Despite repeated condensation cycles, the superhydrophobic characteristic of the fabricated self assembled mono-layer coating did not change, indicating that the superhydrophobic coating was chemically stable. Nevertheless, nanoscale pinholes in the thin coating might cause condensation induced blistering and coating delamination during condensation^58^. As a result, dropwise condensation may fail over a long period of operation. Many studies in recent years have focused on achieving long-term durability for hydrophobic coatings that facilitate dropwise condensation^59–61^. Such potential coatings could be used on the proposed condenser platform to ensure long-term durability.

In practical applications, the proposed wicking reservoir made from layers of filter papers is inadequate and can be interchanged with compressed synthetic fibers or porous ceramics materials that offer better durability. Further research is required to develop a working prototype for microgravity conditions that can be operated for long durations or under high heat flux conditions. In such cases, the proposed condenser platform must be integrated with an external system to continuously transport the collected condensate from the wicking reservoir to a storage tank. The proposed condensate removal mechanism, which utilises patterns of wettability and wicking reservoirs, can also be adapted for high heat flux condensation. This study explored the experiments of water vapour condensation in the presence of air. Therefore, the demonstrated technology is applicable to humidity management within the space station. After filtration and mineralization, the collected condensate from the platform can also be used as drinking water for astronauts.

## Conclusion

In this study, we proposed a proof-of-concept for a novel plate-type condenser platform that facilitates the continuous removal of condensate from a horizontal wettability-patterned surface via a wicking reservoir surrounding the condensing area. This study examined the condensation dynamics and heat transfer performance of four distinct patterns of wettability (P1-P4) comprised of laser-engraved superhydrophilic tracks of varying shapes on a superhydrophobic surface. Using coalescence-driven capillary pumping, the condensate from the superhydrophobic region migrated to the superhydrophilic tracks. The physics of the passive transport of the condensate liquid on various patterns of wettability is investigated using numerical modelling. The Laplace pressure difference of the wedge-shaped superhydrophilic track directs the condensate to the substrate’s side edges. Further, the accumulated condensate along the side edges is absorbed by the wicking reservoir. Flooding of the condensate is observed on the engineered wettability designs of P1 and P2. On the engineered wettability designs P3 and P4, we observed the continuous removal of condensate without flooding. Pattern P4 has demonstrated better condensation heat transfer performance than pattern P3 by more than 15%. Thus, the optimal surface with a patterned wettability can enhance the effective condensation performance. The current research could pave the way for developing efficient condenser surfaces for space applications.

## Methods

### Fabrication of the substrate with patterned wettability and characterization

The substrate material was an aluminium alloy of grade 6061 with dimensions of 40 mm × 40 mm × 3 mm. The substrate was first ultrasonically cleaned for 10 min in a solution of deionized (DI) water, ethanol, and acetone, and then dried with $N_2$ gas. Further, the substrate was microtextured by dipping in 3*M* *HCl* (Merck-EMPLURA grade) for 5 min and washed thoroughly with DI water. The microtextured substrate was immersed in DI water at a temperature of 90 °C for 30 min to grow the boehmite nanostructures above the microstructures^62^. These procedures modified the smooth aluminum substrate to a hierarchical rough superhydrophilic surface with a contact angle of below 5°. Further, the superhydrophilic substrate was functionalized with 0.5 % vol/vol 1H,1H,2H,2H-Perfluoro-octyl-triethoxysilane (PFOTS) in ethanol solution by dip-coating for 3 h followed by drying in a laboratory environment for 12 h ^50^. This procedure transformed the wettability of the substrate from superhydrophilic (SHL) to superhydrophobic (SHB). To fabricate the substrate with patterned wettability, the uniformly coated hydrophobic layer has been selectively removed by laser ablation. A 60 W CO_2_ laser (Universal Laser VLS3.60) having a spot size of 30 μm was used for the laser ablation. The power setting of the laser was 95 % of the total power and the speed setting was 5 % of the total speed. After the laser ablation, the areas that were exposed to the laser changed into SHL, while the areas that weren’t exposed to the laser remained as SHB^21^.

The morphology of the modified hierarchical surface textures was analyzed using a high-resolution scanning electron microscope (Apreo- Thermofisher) after the gold sputtering operation for 30 s. The surface roughness was measured using a surface profilometer (Nanomap 1000WLI). The dynamic contact angles (advancing and receding) were measured by injecting/drawing out the DI water at a flow rate of 0.2 μL to/from a sessile water drop of volume 5 μL using a syringe pump^63^.

### Experimental procedures

**Figure 13:**
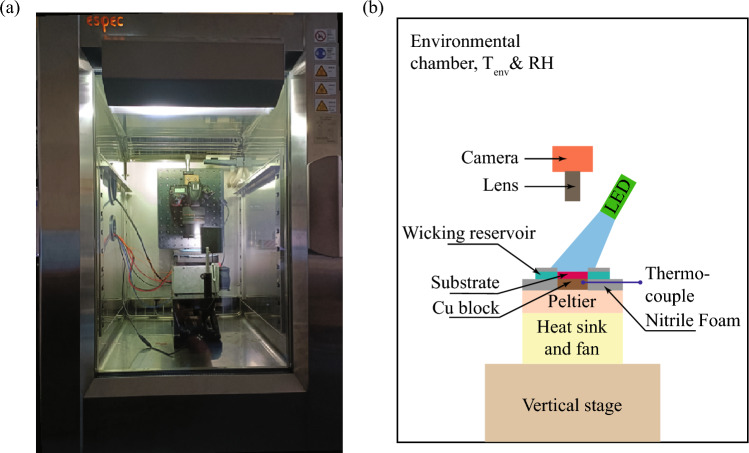
Experimental setup for the condensation experiments on the horizontally oriented substrate with patterned wettability. (**a**) Snapshot and, (**b**) schematic representation.

The condensation experiments on horizontally oriented surfaces were performed in an environmental chamber (Espec-PR2J), as shown in Fig. 13. The variations of temperature and humidity values within the chamber were ±0.5 °C and ±2%. A peltier (TeTech-CP061HT) was used to cool the test substrate, which was placed on a vertical stage in a horizontal orientation. A copper block of dimension 40 mm× 40 mm × 10 mm was attached to the middle of the peltier cold plate using double-sided thermal conductive tape (3M Tape) of a thickness of 0.25 mm. The remaining area of the peltier top plate was covered with a 10 mm thick nitrile foam as thermal insulation. Then, the substrate was attached above the copper block using thermal conductive double-sided tape (3M Tape of 0.25 mm). A wicking reservoir was placed around the substrate to wick the condensate from the substrate. Direct contact between the wicking reservoir and the substrate was avoided to prevent heat transfer loss by conduction, and a gap of 0.5 mm was kept between the substrate and the wicking reservoir (see Fig. 4). Thus, in order to initiate wicking, the liquid-vapor interface of the condensate must cross the gap and reach up to the side edges of the wicking reservoir. The overall condensation performance of the different substrates was measured from the weight difference of the wicking reservoir before and after the condensation experiments in a controlled environment for a duration of 1–2 h. The mass of the wicking reservoir was measured with a precision-balance (Ohaus-SPX622) of 10 mg accuracy. A sheathed T-type thermocouple (Tempsens) of diameter 0.6 mm was inserted through the lateral side of the substrate by making a drilled hole of diameter 0.8 mm. The temperature of the substrate was measured using a data acquisition system (Keysight-970A) at a recording rate of 1 data/min. To obtain the uncertainty in the measurements, each experimental case is repeated multiple times with different surfaces. The time-lapse images of the condensation dynamics on the substrate were recorded at every minutes using a DSLR camera (Nikon-D750) with a zoom lens (24–85 mm Nikon lens), at a focal length of 85 mm.

## Data Availability

The datasets used and/or analysed during the current study available from the corresponding author on reasonable request.
